# Resistance against macrocyclic lactones in *Psoroptes ovis* in cattle

**DOI:** 10.1186/s13071-020-04008-2

**Published:** 2020-03-14

**Authors:** Wouter van Mol, Nathalie De Wilde, Stijn Casaert, Zhenzhen Chen, Marieke Vanhecke, Luc Duchateau, Edwin Claerebout

**Affiliations:** 1 Laboratory of Parasitology, Faculty of Veterinary Medicine, Ghent University, Salisburylaan 133, 9820 Merelbeke, Belgium; 2 Biometrics Research Center, Faculty of Veterinary Medicine, Ghent University, Salisburylaan 133, 9820 Merelbeke, Belgium

**Keywords:** *Psoroptes ovis*, Macrocyclic lactones, Resistance

## Abstract

**Background:**

Psoroptic mange is an important disease in Belgian Blue cattle. Treatment failure of macrocyclic lactones against *Psoroptes ovis* has been reported, but clear evidence of *in vivo* resistance is lacking. This study assessed the efficacy of macrocyclic lactone products on 16 beef farms in Belgium and the Netherlands *in vivo* and *in vitro*.

**Methods:**

On each farm a group of animals (*n* = 7–14) with psoroptic mange was treated with two subcutaneous injections of a macrocyclic lactone product with 7–10 days interval (15 farms) or a single injection with a long-acting macrocyclic lactone (1 farm). *In vivo* efficacy was assessed by the reduction in mite counts, clinical index (proportion of the body surface affected by lesions), the proportion of the animals with negative mite counts after the first treatment round and the number of treatment rounds needed to obtain zero mites counts in all animals. A mite population was categorized as sensitive when the mite count reduction after the first treatment round > 95% and the lower limit of the uncertainty interval > 90%. Resistance was detected when both parameters were below their threshold and suspected when one parameter was too low. *In vitro* knockdown and mortality were evaluated in a contact test.

**Results:**

The proportion of the animals with negative mite counts after the first treatment round varied from 0 to 80%. All farms needed two or more treatments rounds to obtain zero mite counts on all animals. Clinical index only started to reduce after the second treatment round. Mite populations from three farms were categorized as sensitive, one as suspected resistant and 12 as resistant. No correlation was found between *in vitro* lethal dose 50 and knockdown dose 50 values and *in vivo* efficacy parameters.

**Conclusions:**

Unambiguous treatment failure was detected on 12 out of 16 farms, confirming the presence of macrocyclic lactone resistance on Belgian Blue beef farms. *In vitro* parameters could not discriminate the farms based on their *in vivo* sensitivity. The mean reduction in mite counts and the lower limit of the confidence interval are proposed as parameters to identify acaricide resistance.
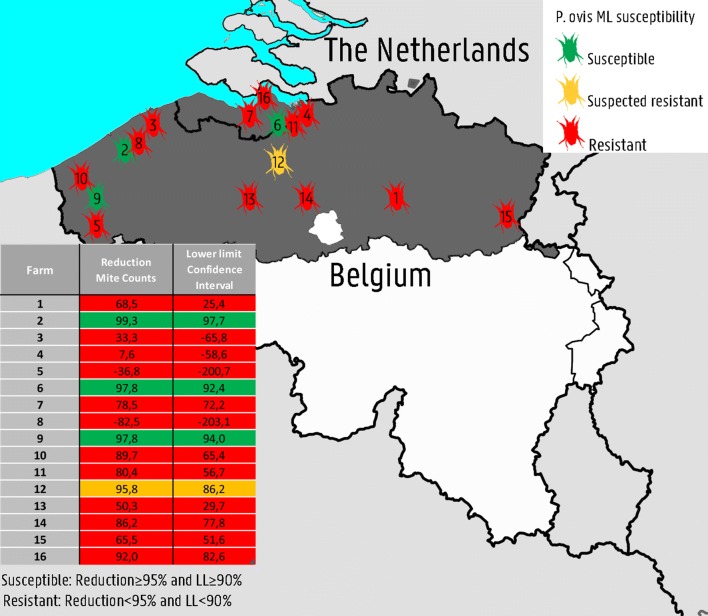

## Background

Psoroptic mange is a major skin problem in sheep and cattle. In cattle, psoroptic mange is predominantly seen in the Belgian Blue breed, due to their high susceptibility to *Psoroptes ovis* [1]. *Psoroptes ovis* infestation causes an exudative perivascular dermatitis with intense pruritus and formation of crusts. The predilection site is from the withers to the tail base. Excessive scratching causes lesions which have a higher chance of secondary bacterial infection. In extreme cases, the lesions can generalize and result in the death of the animal [1–4]. Diminished animal welfare and financial losses are seen on farms with *P. ovis* problems [5, 6].

Registered treatments in Europe are limited to topical acaricides, such as amitraz or synthetic pyrethroids, and parenteral administration of macrocyclic lactones (ML). Topical application of ML products is not recommended, due to contradictory publications about their efficiency to completely eliminate *P. ovis* [7]. In practice, it is recommended to apply acaricides twice with a 7–10-day interval, probably due to a lack of full ovicidal efficacy [7, 8]. Long-acting (LA) ML products only need one application, because of their long persistent activity [8–12]. Additional measures are crucial to get the desired treatment result. The hair needs to be sheared and crusts need to be removed prior to treatment. Treatment should be applied to all in-contact animals and not only the clinically infested animals [3, 7, 11, 12].

Treatment failure with ML products in cattle has been reported by Lekimme et al. [13], Mitchell et al. [14] and Sarre et al. [3]. Potential reasons for treatment failure are non-compliance with the recommended ‘good practice’ treatment and acaricide resistance. Suboptimal treatment cannot be ruled out in the studies mentioned above, as either a long treatment interval (14 days) was used [13] or not all animals were treated in every treatment round [3]. Sarre et al. [15] mentioned that less than half of the Belgian farmers applied correct treatment against *Psoroptes*. Commonly made mistakes include underdosing, a single treatment, a long treatment interval, the use of a suboptimal drug formulation or a lack of additional control measures, e.g. failing to shear the animals or partial treatment of the herd [15]. Recently, decreased *in vitro* sensitivity to ML products was seen in mites collected from sheep farms with reported treatment failure [16, 17]. Lifschitz et al. [18] reported treatment failure in cattle, despite repeated subcutaneous injection of two commercial formulations of ivermectin (1%) at 0.2 mg/kg with a short treatment interval (1 week).

Despite numerous field observations and a few published reports of treatment failure against *P. ovis* in cattle, the presence of resistant mites was not confirmed [3, 13, 18]. The objectives of this study were (i) to investigate the presence of ML resistance in beef cattle in Belgium and The Netherlands using different *in vivo* parameters; and (ii) to evaluate an *in vitro* test based on the method of Brimer et al. [19] as a potential diagnostic tool for ML resistance in *P. ovis.*

## Methods

### Animal selection

Sixteen Belgian Blue beef farms with psoroptic mange were visited during the housing period during the winters of 2016–2017, 2017–2018 and 2018–2019. Skin scrapings were collected from two to five animals per farm to confirm active *P. ovis* infestations.

A group of 7 to 13 animals was selected on each farm. Male or female cattle older than 4 months were used. The experimental group was separated from the rest of the herd and animals in adjacent pens were treated with an acaricide (injectable ML product or topical treatment with amitraz, twice with one-week interval) for the duration of the study, to preclude re-infestation of the study animals.

### Animal treatment

All animals were treated with a commercially available injectable ML product, i.e. ivermectin (Ivomec^®^, Merial, Toulouse, France), doramectin (Dectomax^®^, Zoetis Belgium SA, Louvain-la-Neuve, Belgium) or moxidectin (Cydectin^®^, Zoetis, Girona, Spain) at the recommended dose rate. On one farm, a long-acting formulation of moxidectin (Cydectin 10% LA^®^, Zoetis) was used. The animals were weighed on a calibrated weighing scale to determine the dose prior to treatment. In absence of a weighing scale, heart girth measurements were used to estimate the body weight [20]. All animals were sheared prior to the first treatment administration and crusts were removed as much as possible during shearing. Fresh bedding with or without cleaning of the stable was applied at least once per week.

A treatment round consisted of two injections with a 7–10-day interval or a single injection with a long-acting ML product, as recommended by the manufacturer. Treatment was evaluated 7 days after the second administration or 14 days after administration of a long-acting ML product. If living *P. ovis* were found in one or more animals at a given farm, a new treatment round was started for all the animals in the experiment.

Treatment with ML products was terminated when all animals had negative *P. ovis* mite counts, when the cattle were turned out on pasture at the end of the housing period or if the condition of the animals warranted salvage treatment. In the latter case, animals were treated topically with amitraz (Taktic^®^, MSD Animal Health, Brussel, Belgium) two times with a one-week interval at the recommended dose.

### *In vivo* efficacy

The clinical index (CI) and mite counts (MC) were determined before the first treatment and after every treatment round. The CI is the proportion of the total body surface with clinical lesions. A CI was assigned to all animals by recording the skin lesions (on both sides of the animal) on a silhouette [21].

Skin scrapings from three different locations at the edge of the lesions (9 cm^2^/sampling location) were collected in one tube per animal for MC. If sampling threatened to influence the treatment results (in case of very small lesions), samples were taken from the predilection sites (withers, back, tail basis). Samples were stored at 10 °C and analysed within 24 h after collection to identify and count living *P. ovis* mites using a stereoscopic microscope (magnification 100×). If the mites were too numerous to count, the sample was weighed, 1 g was taken and mites were counted as previously described. Mite numbers were then recalculated to the total mite count of the pooled sample.

### *In vitro* sensitivity

An *in vitro* test was based on Brimer et al. [19]. Sterile polystyrene Petri dishes with an internal diameter of 92 mm were filled with 20 ml sterile agar solution (Bidest with 42 g/litre Columbia agar, Sigma-Aldrich, St. Louis, USA) containing 1 ml horse serum (food source) and 0, 1, 10, 100, 1000, 2000 and 5000 µg/ml ivermectin (1% Ivomec^®^, Boehringer-Ingelheim).

The *in vitro* sensitivity was determined on mites from pooled skin scrapings from each farm. Ten mites (adults and nymphs from Farms 1–10, adult mites form Farms 11–16) from each pooled sample were transferred to the centre of the Petri dish. The plates were kept at room temperature for 24 h. The mites were inspected under a stereomicroscope at 0, 2, 4, 6, 8 and 24 h of incubation. Immobility of mites and a lack of reactions or persistent immobility within 1 min following stimulation with a needle were considered indications of death. All concentrations were tested in triplicate, except for Farms 1 and 8. The test for Farm 1 was conducted *in duplo* and without the 5000 µg/ml ivermectin plates. This concentration was added afterwards for the other farms, as 50% mortality was not achieved with lower concentrations on Farm 1. Farm 8 was tested *in duplo*, due to a shortage of mites.

Additional time points and an extra parameter (‘knockdown’) were monitored for Farms 11–16. The status of the mites was recorded at 0, 5 min, 0.5, 1, 2, 4,6, 8, 24, 48, 72 and 120 h of incubation. Knockdown was defined as the absence of spontaneous mobility, while movement was still observed after stimulation with a needle.

### Statistical analysis

#### In vivo efficacy

Multiple parameters were monitored *in vivo*, i.e. number of treatment rounds until all animals at a given farm had a mite count of zero (nTR), reduction in MC after first treatment round (RedMC), CI reduction after two treatment rounds (RedCI) and the proportion of the animals with zero mite counts after the first treatment round.

RedMC is defined as

$${\text{RedMC }}\left( \% \right) = 100 \left( {1 - \frac{{\overline{\text{MCA}} }}{{\overline{\text{MCB}} }}} \right)$$

where $\overline{\text{MCB}}$ and $\overline{\text{MCA}}$ is the mean MC value of a number of cows before and after the first treatment round, respectively, with its variance approximated by the delta method as

$${\text{Var}}\left( {\text{RedMC}} \right) = \left( {\frac{{\overline{\text{MCA}} }}{{\overline{\text{MCB}} }}} \right)^{2} \left( {\frac{{{\text{Var}}\left( {\overline{\text{MCA}} } \right)}}{{\overline{\text{MCA}} }} + \frac{{{\text{Var}}\left( {\overline{\text{MCB}} } \right)}}{{\overline{\text{MCB}} }} - 2 \frac{{{\text{Corr}}\left( {\overline{\text{MCA}} ,\overline{\text{MCB}} } \right)\sqrt {{\text{Var}}\left( {\overline{\text{MCA}} } \right) {\text{Var}}\left( {\overline{\text{MCB}} } \right)} }}{{\overline{\text{MCA}} \overline{\text{MCB}} }}} \right)$$

Assuming that 100-Red_MC_ follows a gamma distribution with shape parameter γ and scale parameter θ given by

$$\gamma = \frac{{\left( {1 - {\text{RedMC}}} \right)^{2} n}}{{{\text{Var}}\left( {\text{RedMC}} \right)}}, \theta= \frac{{{\text{Var}}\left( {\text{RedMC}} \right)}}{{\left( {1 - {\text{RedMC}}} \right)^{2} n}}$$

the lower limit (LL) and upper limit (UL) of the 95% confidence interval correspond to the 2.5th and the 97.5th quantile of the gamma distribution with shape parameter γ and scale parameter θ.

The RedMC and its LL and UL where then calculated for each farm. Calculations were done with the Paradrug tool^®^ (www.starworms.org/tools). Mite isolates of a farm were then categorized according to the methodology used for the faecal egg count reduction test in gastrointestinal nematodes, with the highest sensitivity [22]. The mite population on a farm was considered sensitive when RedMC ≥ 95% and LL ≥ 90%, suspected of resistance when RedMC < 95% or LL < 90% and resistant when RedMC < 95% and LL < 90%.

The proportion of the animals with negative mite counts was the fraction that was successfully treated (MC = 0) after the first treatment round.

#### In vitro sensitivity

The lethal dose 50 (LD_50_) values for each farm population was calculated for the survival at 24 h and 120 h and knockdown dose 50 (KD_50_) values at 24 h using a mixed logistic regression model with mortality and knockdown as binary response variable, drug concentration as continuous independent variable and farm as random effect.

Spearmanʼs rank correlations were calculated between nTR, RedMC, RedCI, mite free proportion and log(LD_50_). Regression analysis was done by fitting a linear model with nTR, mean Red_MC_, mean Red_CI_ or the proportion of the animals with negative mite counts as independent variable and log(LD_50_) as dependent variable. All calculations were done in R (version 3.5.1.) [23].

## Results

### Participating farms

The farms were located in Flanders, northern Belgium, (*n* = 13) and the Zeeland province of The Netherlands, bordering Belgium (*n* = 3). Group sizes varied from 7 to 13 animals/farm. The injectable ML products used were ivermectin (Ivomec^**®**^1%, *n* = 5), doramectin (Dectomax^**®**^, *n* = 11), moxidectin (Cydectin^**®**^ 1 %, *n *= 1) and moxidectin long-acting (Cydectin 10% LA^**®**^, *n* = 1). One farmer switched from ivermectin to doramectin after the third injection. Moxidectin long-acting was only used on one farm during the first treatment round. Afterwards, the farmer used doramectin.

### *In vivo* efficacy

**Fig. 1:**
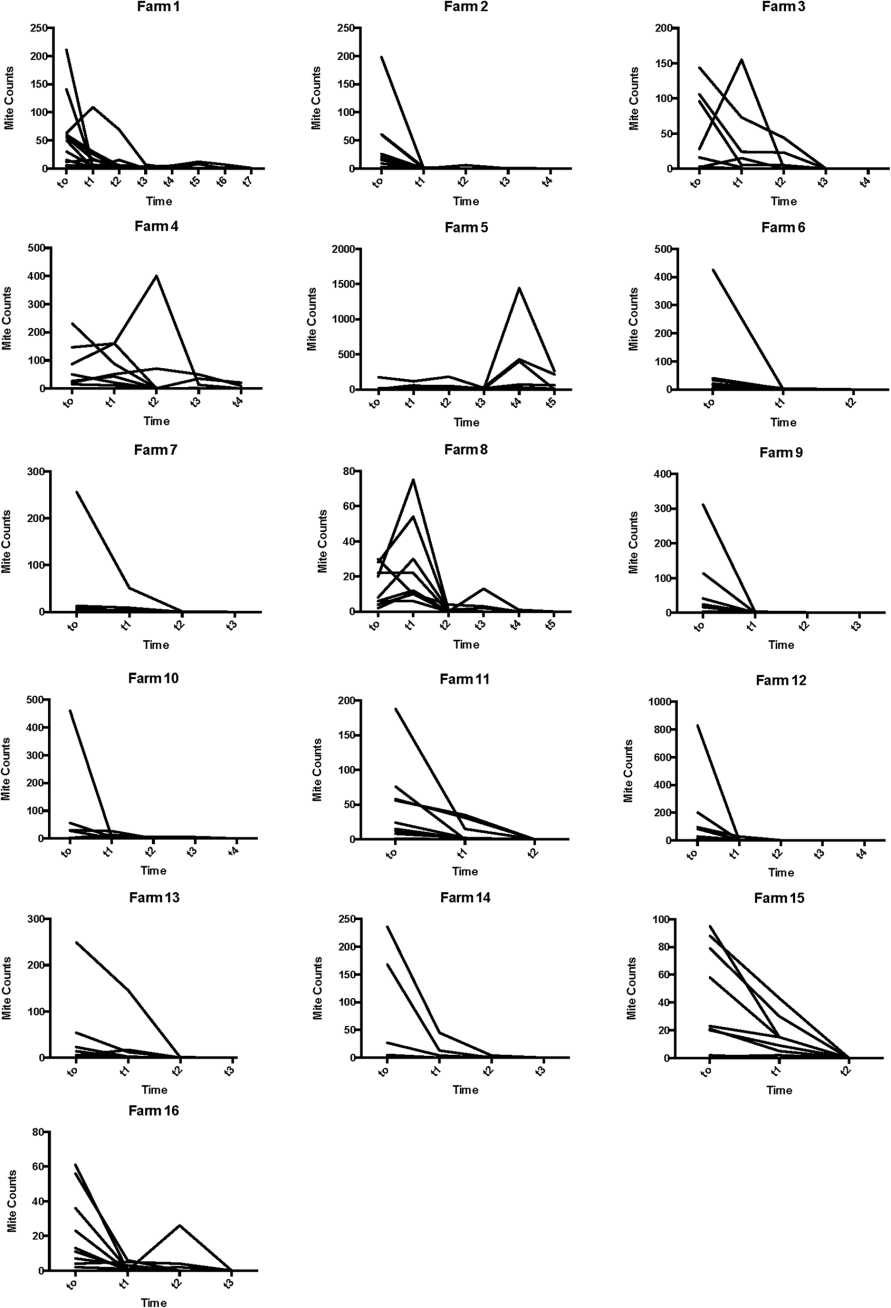
The individual mite counts of each animal (sum of the three pooled samples) are depicted for the 16 farms. MC are given on the y-axis and time in number of treatment rounds on the x-axis. t0 refers to the count prior to the initiation of the first treatment round, t1 is after the first treatment round, etc. Each line depicts MC values of one animal over time

All farms needed at least two treatment rounds and one farm even up to 6 rounds, to count zero mites in all animals at a given farm. The proportion of the animals with negative mite counts after the first treatment round varied between 0–80%. The individual mite counts per animal are shown in Fig. 1.

**Fig. 2:**
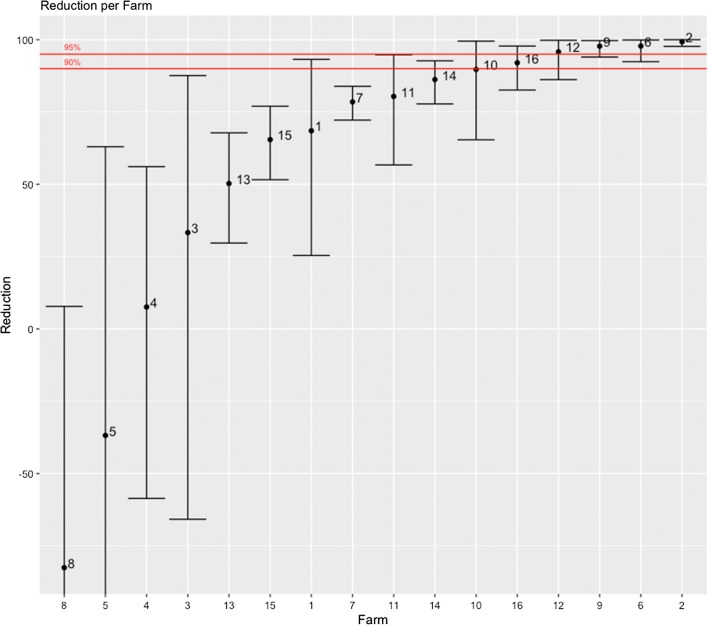
RedMC is depicted for the mite populations of the given farms. The uncertainty intervals are given by the whiskers. The 90% and 95% threshold are indicated by red lines

RedMC varied from − 82.5% to 99.3% (Table 1). The RedMC and the corresponding uncertainty interval of the mite populations on all the given farms are illustrated in Fig. 2. Treatment with ML products was terminated on four farms before all animals of those farms had a mite count of zero, due to unsatisfying treatment results (Farms 2, 4 and 5) or the end of the housing period approached (Farm 1). These groups were successfully treated with amitraz (results not shown).

**Table 1:** *In vivo* efficacy of macrocyclic lactone products against *Psoroptes ovis* on 16 Belgian Blue beef farms in Belgium and the Netherlands

Farm	No. of animals	Acaricide	RedMC (%)	95% CI	Resistance status	nTR	Mite-free proportion (%)
1	13	IVM	68.5	25.4–93.2	Resistant	> 7	0/13 (0)
2	10	IVM	99.3	97.7–100	Sensitive	4	8/10 (80)
3	9	IVM /DOR	33.3	− 65.8 to 87.6	Resistant	3	2/9 (22)
4	7	MOX-LA DOR	7.6	− 58.6 to 56.1	Resistant	> 4	0/7 (0)
5	9	IVM	− 36.8	− 200.7 to 63.0	Resistant	> 5	0/7 (0)
6	9	DOR	97.8	92.4–99.0	Sensitive	2	4/9 (44)
7	9	DOR	78.5	72.2–83.9	Resistant	4	2/9 (22)
8	9	IVM	-82.5	− 203.1 to 7.8	Resistant	5	2/9 (22)
9	10	DOR	97.8	94.0–99.7	Sensitive	3	5/10 (50)
10	9	MOX	89.7	65.4–99.5	Resistant	4	2/9 (22)
11	9	DOR	80.4	56.7–94.8	Resistant	2	3/9 (33)
12	9	DOR	95.8	86.2–99.8	Suspected resistant	4	1/9 (11)
13	8	DOR	50.3	29.7–67.8	Resistant	3	4/8 (50)
14	8	DOR	86.2	77.8–92.7	Resistant	3	5/8 (63)
15	10	DOR	65.5	51.6–77.0	Resistant	2	2/10 (20)
16	9	DOR	92.0	82.6–97.8	Resistant	3	3/9 (33)

*Abbreviations*: CI, confidence interval; IVM, ivermectin injectable (Ivomec 1%^®^); DOR, doramectin injectable (Dectomax^®^); MOX, moxidectin injectable (Cydectin^®^); LA, moxidectin injectable long-acting formulation; RedMC, average reduction in mite counts on a given farm after the first treatment round; nTR, number of treatment rounds until all animals at a given farm had a mite count of zero; mite-free proportion, the proportion of the animals with negative mite counts after the first treatment round

**Fig. 3:**
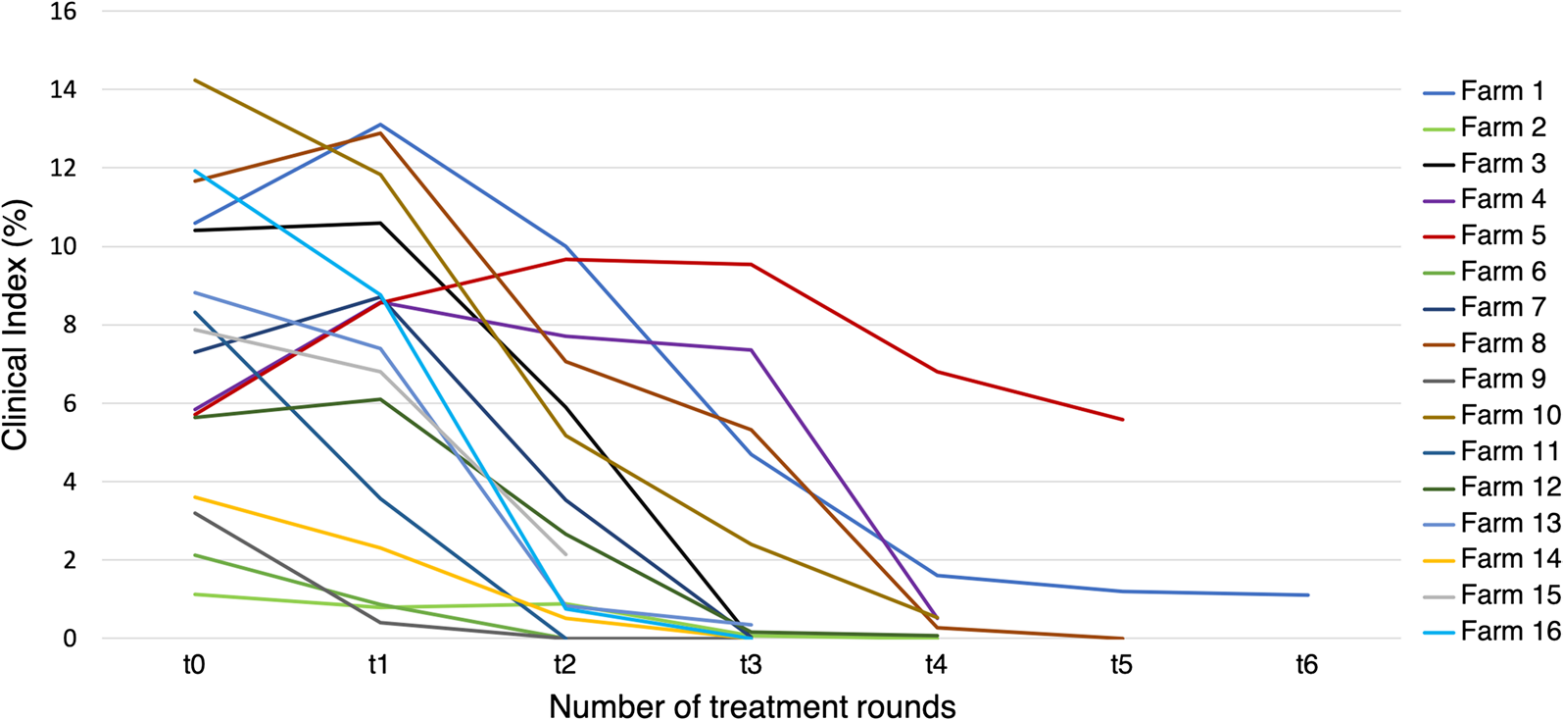
The progress of the clinical index for the animals on each farm. t0 refers to the clinical index prior to the first treatment round, t1 is the clinical index after the first treatment round, t2 is the clinical index after the second treatment round … t6 is the clinical index after the sixth treatment round

The evolution of the CI of the animals on the different farms is depicted in Fig. 3. The mean CI before treatment varied between 1–14%. On most farms, the skin lesions were completely healed (CI = 0) at the end of the treatments. Farms 1, 4 and 10 had a mean CI of 1% at the end of the treatment. Only Farm 5 did not show any overall improvement of the CI during treatment.

### *In vitro* sensitivity

The LD_50_ values at 24 h in mite populations from Farm 1 to 16 varied from 2951 µg/ml to 94,499 µg/ml with some populations not even reaching 50% mortality. The additional time point of 120 h for Farm 11 to 16 provided LD_50_ values from 0.3 µg/ml to 58.3 µg/ml (Table 2).

**Table 2:** LD_50_ at 24 h and at 120 h and KD_50_ at 24 h for ivermectin in *P. ovis* populations from 16 Belgian Blue farms in Belgium and the Netherlands

Farm	LD_50_; 24 h (µl/ml)	LD_50_; 120 h (µl/ml)	KD_50_; 24 h (µl/ml)
1	36867	na	na
2	2951	na	na
3	2992	na	na
4	3972	na	na
5	3417	na	na
6	3430	na	na
7	4172	na	na
8	3121	na	na
9	3175	na	na
10	3304	na	na
11	6.538 × 10^6^	23.1	0.19
12	11.337	2.7	0.08
13	3191	0.3	0.18
14	7429	58.3	0.05
15	6857	10.4	0.33
16	94499	3.6	0.77

*Abbreviation*: na, data not available

KD_50_ were determined for Farm 11 to 16 and varied from 0.05 to 0.77 µg/ml ivermectin at 24 h. Neither LD nor KD allowed for a clear discrimination of the mite populations of the cattle of the 16 farms.

The regression analysis showed no correlation between *in vivo* parameters and *in vitro* LD_50_ values. Spearmanʼs rank correlations were low (− 0.28–0.36) and *P*-values were too high (0.338–0.730).

## Discussion

When ML products were introduced in the market, injectable ML products were highly effective against *P. ovis.* A single administration of the recommended (commercialised) dose of an injectable ML product was sufficient to kill the mites and obtain clinical cure [24–28]. In practice, a second injection is recommended to get the desired efficacy [7, 25].

In the present study, two or more treatments with weekly intervals were insufficient to eliminate *P. ovis*. Treatment failure of ML products against *P. ovis* has been reported in sheep and cattle [3, 13, 16, 18]. Although treatment failure can often be attributed to non-compliance with recommended treatment and control measures [15], resistance of *P. ovis* populations against ML is suspected as a cause of treatment failure [16–18] and could be the result of decades of frequent and indiscriminate use of ML.

In this study, confounding factors that could interfere with the detection of ML resistance were eliminated as much as possible. On all farms, the experimental animals were treated according to ‘good practice’, including weighing/girth measurements to determine the dose rate, use of an injectable formulation and treatment of the whole group with appropriate treatment interval. Prior to treatment, crusts were removed manually and the animals were sheared, to optimise treatment efficacy. Despite these efforts, poor (or no) mite count reductions were obtained after the first treatment round and multiple treatment rounds were needed to eliminate the mites and to obtain healing of the skin.

Although ML concentrations in the skin were not determined, it seems unlikely that treatment failure was due to low ML concentration in the skin of the treated animals, given the high number of treatments with short time intervals. Lifschitz et al. [18] showed high skin levels of ivermectin in cattle after two injections with a seven-day interval. Although BB cattle are more susceptible to psoroptic mange than other breeds, ML concentrations in the plasma and skin of BB were actually higher than in Holstein-Friesian animals [29]. Based on the above, the observed treatment failure in the present study is highly indicative for ML resistance.

The reduction in MC after the first treatment round appeared to be the best parameter to discriminate between sensitive and resistant mite populations, as MC reductions directly measure the effect of the ML on the population of mites. The number of treatment rounds and the proportion of the animals with negative mite counts after the first treatment round did not discriminate between populations, although they are derived from the reduction in MC. CI failed to differentiate between populations after treatment, since clinical recovery requires more time than mite eradication. This is also observed in ML sensitive mites [12].

To date, no threshold for mite count reductions has been defined to distinguish sensitive from resistant mite populations *in vivo*. We have based our classification of resistance on the methodology with highest sensitivity to detect anthelmintic resistance in ruminant nematodes using a faecal egg count reduction test [22, 30]. Using the LL of the UI and the RedMC described by Coles et al. [30], we identified three farms with sensitive mites, one farm was classified as ‘suspected resistance’ and 12 farms had resistant mite populations. This methodology was used because of the lack of resistance guidelines for ectoparasites in cattle. The World Association for the Advancement of Veterinary Parasitology (WAAVP) and the European Medicines Agency do have guidelines for the efficacy of new acaricides, recommending MC as efficacy parameter and a 90% mean reduction in MC after treatment as sufficient [31, 32]. Compared with these guidelines for acaricide efficacy, the thresholds we used to distinguish sensitive from resistant mite isolates may seem to be harsh. However, survival of 10% of the mites could quickly result in a relapse of psoroptic mange in the treated animals, as the mite population can double in size every six days under favourable conditions [33]. Moreover, even if the WAAVP efficacy guidelines for acaricide efficacy would have been followed, ML efficacy would only be sufficient on 5 out of 16 farms (MC reduction > 90%), which supports our conclusion that ML resistance is widespread in BB farms in northern Belgium and the south of the Netherlands. The presence of ML resistance in Belgium is no surprise, since compliance with the recommended treatment is poor in the majority of Belgian beef farmers [15]. The emergence of ML resistance in *P. ovis* in beef farms in different countries (Argentina, Belgium, the Netherlands) is concerning and needs further investigation. Beef farmers should be instructed about good practices in mange control to slow down further spreading and development of resistance. Research is ongoing to identify the factors that may impede or facilitate adoption of these practices among farmers [34].

An *in vitro* test based on exposure of mites to different concentrations of acaricides on agar plates [19] has been used to differentiate sensitive and resistant mites [16]. An *in vitro* test would be useful to determine the resistance status of mite populations without the need to perform a labour-intensive mite count reduction test. However, in our hands, the *in vitro* test failed to distinguish the mite populations with different *in vivo* sensitivity from each other and *in vitro* test results did not correlate with *in vivo* efficacy. Despite large differences in MC reductions and CI reductions between farms, the *in vitro* efficacy showed a similar pattern in all farms and LD_50_ values or KD_50_ values did not vary with *in vivo* efficacy. The reason for this is unknown. To our knowledge, data about the reproducibility between laboratories are not available [19, 22].

## Conclusions

Unambiguous treatment failure of ML products was detected on 12 out of 16 beef farms, confirming the presence of ML resistance in Belgian Blue beef farms in Belgium and the Netherlands. Tentative *in vitro* parameters could not detect ML resistance. The reduction in mite counts after the first treatment round (two administrations of an injectable ML product) appears to be a suitable parameter to monitor acaricide resistance. A mean reduction in mite counts of < 95% combined with a lower limit of the 95% uncertainty interval < 90% are proposed as criteria for the detection of resistance against ML in *P. ovis.*

## Data Availability

Data supporting the conclusions of this article are included within the article. The datasets used and/or analysed during the present study are available from the corresponding author upon reasonable request.

## Abbreviations

ML: macrocyclic lactones
LA: long-acting
CI: clinical index
MC: mite counts
nTR: number of treatment rounds
RedMC: reduction in mite counts
RedCI: reduction in clinical index
$\overline{\text{MCB}}$: mite counts before the first treatment round
$\overline{\text{MCA}}$: mite counts after the first treatment round
LL: lower limit of the confidence interval
LD_50_: lethal dose 50
KD_50_: knockdown dose 50
IVM: ivermectin
DOR: doramectin
MOX: moxidectin

## References

[1] Losson BJ, Lonneux JF, Lekimme M (1999). The pathology of *Psoroptes ovis* infestation in cattle with a special emphasis on breed difference. Vet Parasitol.

[2] Pruett JH, Guillot FS, Fisher WF (1986). Humoral and cellular immunoresponsiveness of stanchioned cattle infested with *Psoroptes ovis*. Vet Parasitol.

[3] Sarre C, Geurden T, Vercruysse J, De Wilde N, Casaert S, Claerebout E (2015). Evaluatie van twee intensieve behandelingsschema’s tegen Psoroptes ovis-schurft bij Belgisch witblauwe runderen op negen Vlaamse rundveebedrijven. Vlaams Diergeneeskd Tijdschr.

[4] van den Broek AH, Huntley JFÃ (2003). Sheep scab: the disease, pathogenesis and control. J Comp Pathol.

[5] Rehbein S, Visser M, Meyer M, Lindner T (2016). Ivermectin treatment of bovine psoroptic mange: effects on serum chemistry, hematology, organ weights, and leather quality. Parasitol Res.

[6] Lonneux JF, Nguyen TQ, Detry J, Farnir F, Losson BJ (1998). The relationship between parasite counts, lesions, antibody titres and daily weight gains in *Psoroptes ovis* infested cattle. Vet Parasitol.

[7] Vercruysse J, Rew RS, Vercruysse J, Rew RS (2002). The use of macrocyclic lactones to control parasites of cattle. Macrocyclic lactones in antiparasitic therapy.

[8] Lekimme M, Mignon B, Leclipteux T, Tombeux S, Marechal F, Losson B (2006). *In vitro* tests for evaluation of the hatchability of the eggs of *Psoroptes* mites following exposure to acaricidal compounds. Med Vet Entomol.

[9] Bridi AA, Carvalho LA, Cramer LG, Barrick RA (2001). Efficacy of a long-acting formulation of ivermectin against *Psoroptes ovis* (Hering, 1838) on cattle. Vet Parasitol.

[10] Lifschitz A, Virkel G, Ballent M, Sallovitz J, Imperiale F, Pis A (2007). Ivermectin (3.15%) long-acting formulations in cattle: absorption pattern and pharmacokinetic considerations. Vet Parasitol.

[11] O’Brien DJ (1999). Treatment of psoroptic mange with reference to epidemiology and history. Vet Parasitol.

[12] Lonneux JF, Nguyen TQ, Losson BJ (1997). Efficacy of pour-on and injectable formulations of moxidectin and ivermectin in cattle naturally infected with *Psoroptes ovis*: parasitological, clinical and serological data. Vet Parasitol.

[13] Lekimme M, Farnir F, Maréchal F, Losson B (2010). Failure of injectable ivermectin to control psoroptic mange in cattle. Vet Rec.

[14] Mitchell ES, Jones JR, Foster AP, Millar M, Milnes A, Williams J (2012). Clinical features of psoroptic mange in cattle in England and Wales. Vet Rec.

[15] Sarre C, De Bleecker K, Deprez P, Levecke B, Charlier J, Vercruysse J (2012). Risk factors for *Psoroptes ovis* mange on Belgian Blue farms in northern Belgium. Vet Parasitol.

[16] Doherty E, Burgess S, Mitchell S, Wall R (2018). First evidence of resistance to macrocyclic lactones in *Psoroptes ovis* sheep scab mites in the UK. Vet Rec.

[17] Sturgess-Osborne C, Burgess S, Mitchell S, Wall R (2019). Multiple resistance to macrocyclic lactones in the sheep scab mite *Psoroptes ovis*. Vet Parasitol.

[18] Lifschitz A, Fiel C, Steffan P, Cantón C, Muchiut S, Dominguez P (2018). Failure of ivermectin efficacy against *Psoroptes ovis* infestation in cattle: integrated pharmacokinetic-pharmacodynamic evaluation of two commercial formulations. Vet Parasitol.

[19] Brimer L, Bønløkke L, Pontoppidan C, Henriksen SA, Gyrd-Hansen N, Rasmussen F (1995). A method for *in vitro* determination of the acaricidal effect of ivermectin using *Sarcoptes scabiei* var. *suis* as test organism. Vet Parasitol.

[20] Fiems L (2004). Gewicht bij witblauwe dikbillen. Veeteelt Vlees.

[21] Guillot FS (1981). Population increase of *Psoroptes ovis* (Acari: Psoroptidae) on stanchioned cattle during summer. J Med Entomol.

[22] Levecke B, Kaplan RM, Thamsborg SM, Torgerson PR, Vercruysse J, Dobson RJ (2018). How to improve the standardization and the diagnostic performance of the fecal egg count reduction test?. Vet Parasitol.

[23] R Core Team (2017). R: a language and environment for statistical computing. Vienna: R Foundation for Statistical Computing. https://www.R-project.org/.

[24] Clymer BC, Janes TH, McKenzie ME (1997). Evaluation of the therapeutic and protective efficacy of doramectin against psoroptic scabies in cattle. Vet Parasitol.

[25] Lonneux JF, Nguyen TQ, Delhez M, Losson BJ (1997). Antibody response after treatment in cattle infected with *Psoroptes ovis*. Res Vet Sci.

[26] Guillot FS, Meleney WP (1982). The infectivity of surviving *Psoroptes ovis* (Hering) on cattle treated with ivermectin. Vet Parasitol.

[27] Lonneux JF, Losson BJ (1992). Field efficacy of injectable and pour-on moxidectin in cattle naturally infested with *Psoroptes ovis* (Acarina: Psoroptidae). Vet Parasitol.

[28] Logan NB, Weatherley AJ, Phillips FE, Wilkins CP, Shanks DJ (1993). Spectrum of activity of doramectin against cattle mites and lice. Vet Parasitol.

[29] Vercruysse J, Deprez P, Everaert D, Bassissi F, Alvinerie M (2008). Breed differences in the pharmacokinetics of ivermectin administered subcutaneously to Holstein and Belgian Blue calves. Vet Parasitol.

[30] Coles GC, Bauer C, Borgsteede FHM, Geerts S, Klei TR, Taylor MA (1992). World Association for the Advancement of Veterinary Parasitology (WAAVP) methods for the detection of anthelmintic resistance in nematodes of veterinary importance. Vet Parasitol.

[31] Vercruysse J, Rehbein S, Holdsworth PA, Letonja T, Peter RJ (2006). World Association for the Advancement of Veterinary Parasitology (WAAVP) guidelines for evaluating the efficacy of acaricides against (mange and itch) mites on ruminants. Vet Parasitol.

[32] Committee for medicinal products for veterinary use. In: Guideline on specific efficacy requirements for ectoparasiticides in cattle. 2005. https://www.ema.europa.eu/en/documents/scientific-guideline/guideline-specific-efficacy-requirements-ectoparasiticides-cattle_en.pdf. Accessed 4 Aug 2019.

[33] Wall R, Shearer D, Wall R, Shearer D (2001). Chapter 2. Mites. Veterinary ectoparasites: biology, pathology, and control.

[34] Mingolla C, Hudders L, Vanwesenbeeck I, Claerebout E (2019). Towards a biased mindset: an extended theory of planned behaviour framework to predict farmers’ intention to adopt a sustainable mange control approach. Prev Vet Med.

